# The Great Wanderer: The Phylogeographic History of the Bicolor Pyramid Ant (*Dorymyrmex bicolor* Wheeler, 1906) in Central Veracruz, Mexico

**DOI:** 10.3390/insects16080785

**Published:** 2025-07-31

**Authors:** Maria Gómez-Lazaga, Alejandro Espinosa de los Monteros

**Affiliations:** Departamento de Biología Evolutiva, Instituto de Ecología AC, Carretera Antigua a Coatepec 351, Xalapa, Veracruz 91070, Mexico; maria.glazaga@gmail.com

**Keywords:** biogeography, Formicidae, genealogy, recent Ne expansion, restricted gene flow, incomplete lineage sorting

## Abstract

The goal of phylogeography is to explain how microevolutionary forces shape the gene pool of a lineage into the geography. In this study we have evaluated the amount of genetic variation in 13 populations of *Dorymyrmex bicolor* distributed in a mountainous region in Central Veracruz, Mexico. To do so, we sequenced fragments from the mitochondrial *COI*, *COII*, and nuclear *LWRh* genes. Segregated sites were found only at the mitochondrial markers, recovering a total of 21 different haplotypes. The nucleotide diversity ranged from 0 to 0.5% at the different sampling sites. Phylogenetic and spatial analyses of molecular variance revealed a weak but significant phylogeographic structure associated with lowland and mountainous zones. Molecular clock analysis suggests that radiation in the mountain area started 7500 years ago, whereas lineage radiation in the lowland started more recently, around 2700 years ago. The phylogeographic structure is incipient, with nests from lowlands more closely related to mountain nests than to other lowland nests, and vice versa. This seems to be consistent with a model of incomplete lineage sorting. The obtained patterns appear to be the result of restricted gene flow mediated by a complex topographic landscape that has been shaped by a dynamic geologic history.

## Simple Summary

The process related to the cohesion of populations within a species depends, at least in part, on the possibility of individuals from one population migrating into a different one and blending their genes by interbreeding with mates from the host population. The bicolor pyramid ant is a small insect, 2 mm in length, distributed from the southwestern United States to Peru. It has wide ecological adaptability and several functional roles. The species is found from sea level to the mountains up to 1500 m, mainly in arid grasslands, but it can be easily found in temperate and tropical forests. This ant presents a high tolerance to disturbance, inhabiting areas altered by human actions. In this study we have evaluated the amount of genetic variation contained in 13 populations of this species distributed in a mountainous region at Central Veracruz, Mexico. Our findings suggest that the local sierras represent emerging barriers to the dispersal abilities of this species. Some individuals, however, have an aptitude for clearing such barriers and dispersing their genes to other populations. Such imperfect barriers, along with a complex geologic history, have produced a mosaic where genetic variation has been arranged in an incipient altitudinal geographic pattern.

## 1. Introduction

Phylogeography, the study uniting population genetics, evolutionary history, and the geographic factors explaining genetic diversity, has been fundamental in determining the processes that create and maintain biodiversity [1,2]. This discipline attempts to explain the forces and processes that shape the geographic distribution of lineages. Understanding how genetic variations within a species change over space and time is essential for determining its evolutionary history. Phylogeographic processes, therefore, are at least in part responsible for the origin of new species and for evolution above the species level. In insects, phylogeographic research has revealed complex divergence patterns shaped by physical barriers, climatic history, and dispersal ability, enabling inferences about population structure, speciation, and helping to uncover cryptic species [2]. Phenotypic specialization, reproductive system, and dispersal capacity have a strong influence on patterns of genetic differentiation. For example, in cave-dwelling orthopterans, variation in the dependence on subterranean ground features helps explain variation in the depth of genetic divergence among co-distributed groups [3]. Molecular analyses showed considerable genetic divergence between the different populations of these species and significant structuring across caves. In ants, phylogeographic studies on *Atta cephalotes* populations have contributed to uncovering the influence of large-scale barriers, such as the Andes, in constraining gene flow and population differentiation [4].

The Central Veracruz region lies between the Sierra Madre Oriental and the Trans-Mexican Volcanic Belt [5]. This region is distinguished by its extremely rugged topography and abrupt altitudinal gradient, spanning from sea level to over 5500 m.a.s.l., all within 100 km. This orographic heterogeneity results in a great variety of climate and vegetation types, ranging from dry environments with elevated temperatures, such as tropical semi-deciduous forests and tropical oak forests, to humid temperate zones with pine–oak and humid montane forests, and even to cold and dry conditions at higher elevations where pine and fir forests predominate [6]. The interaction of tropical and temperate atmospheric systems, the proximity to the Gulf of Mexico, and sharp elevation changes result in contrasting microclimates across the region. Biodiversity fluctuations result from processes of speciation and genetic differentiation due to these environmental gradients [7]. Additionally, Central Veracruz is the most populated region of the state. Anthropogenic factors definitely have an effect on population dynamics and in shaping biodiversity. Land transformation, species translocation, and the annihilation of local populations have a strong effect on modifying genetic structure. Alternatively, habitat fragmentation and landscape transformation might facilitate the dispersion of generalist invasive species. For all these reasons, Central Veracruz is an important area to analyze phylogeographic patterns and evolutionary mechanisms in insects and other organisms. Recently, several phylogeographic studies in co-distributed insect taxa have been published, including some for the dung beetle *Canthon cyanellus*, where results revealed the presence of deeply divergent lineages shaped by Pleistocene climatic fluctuations and geological barriers, such as volcanic fields and elevation gradients [8]. Similarly, the staphylinid beetle *Falagonia mexicana* showed a robust genetic structure associated with geological events and altitudinal restrictions [9]. *Falagonia mexicana* and other insect species appear to represent biological systems showing swift geographic range expansions, followed by subsequent restricted gene flow mediated by the intricate mountainous regions of Mexico.

*Dorymyrmex bicolor* is a small ant species, approximately 2 mm in length, belonging to the subfamily Dolichoderinae. The combination of single segmented petiole, mesosomal profile with two well-developed tubercles and body concolorous light reddish brown to yellowish with pubescence sparse can identify this species of any other ant. The bicolor pyramid ant does not have the ability to sting. Despite of its short size they can run fast, and during dry days are active from early to late afternoon. During daytime the workers show a vigorous foraging behavior. As many other ants, this one is considered a good seed dispersal insect strongly affecting germination and plant distribution. Its nest is easy to spot and identify by its crater-shaped exit made of fine grains of sand forming a circle of nearly 100 mm in diameter (mean = 104.12, SD = 21.38, n = 20; Supplementary Material Figure S1). It is distributed in a wide range of territory from the southwestern United States to Peru. It is a dominant species with wide ecological adaptability and functional roles. It has been documented in various contexts as desert grasslands, disturbed areas in arid valleys, peri-urban areas, and even a tropical rainforest [9,10,11,12]. Similar to other *Dorymyrmex* species, the bicolor pyramid ant presents a high tolerance to disturbance; being found both in degraded habitats and cattle-grazing land [11] and is frequently found in sandy soils colonized by herbaceous vegetation [9]. It also uses different foraging strategies, including scavenging [13], extrafloral nectar consumption, and mutualistic interactions with hemipterans [14]. Despite its wide distribution and ecological versatility, the evolutionary history and species delimitation of *Dorymyrmex bicolor* remain poorly understood. Recent phylogenomic studies have shown that the genus *Dorymyrmex* has a complex amphitropical distribution and might include several morphologically cryptic lineages [15,16]. Apparently, this genus has experienced a recent evolutionary radiation with little morphological differentiation.

This taxonomic uncertainty, combined with its broad ecological amplitude and capacity for dispersal via winged reproductives, makes *D. bicolor* an excellent candidate for biogeographic and phylogeographic studies at both continental and regional scales. Although this study focuses on populations from Central Veracruz, the region’s geological and ecological heterogeneity provides a natural framework to explore genetic structure, potential barriers to gene flow, and signs of local differentiation within a widespread and generalist ant species. This study aims to analyze the genetic diversity and population structure of *Dorymyrmex bicolor* in Central Veracruz.

## 2. Materials and Methods

### 2.1. Collection Sites

**Figure 1:**
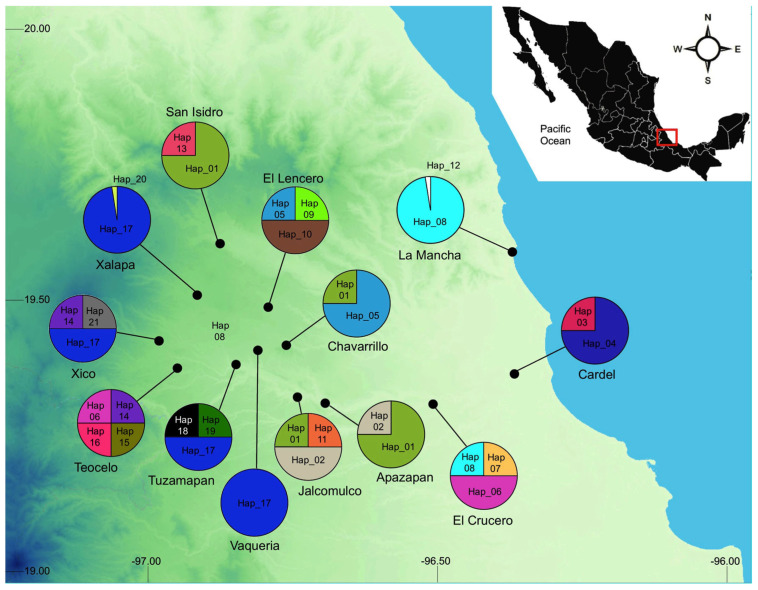
Collection sites, haplotype distribution, and local frequency. Hap_17 showed the widest distribution. It was found above 800 m in 4 cloud forest sites and in 12 different nests.

Populations of *Dorymyrmex bicolor* were sampled from 13 localities in the central region of Veracruz, Mexico (Figure 1, Table 1). The collection sites were selected based on field observations, collection records, and local knowledge regarding the presence of the species. Broadly, these localities can be divided according to their vegetation type and weather as follows: (1) In La Mancha, the main habitat are coastal dunes with mangrove forest and seasonal wetlands, the annual mean temperature is 24 °C, and it rains all year, with peak precipitation during summer. (2) Apazapan, Cardel, El Crucero, and Jalcomulco are located below 500 m a.s.l.; the main vegetation type in these sites is tropical deciduous forests; the annual mean temperature is 24 °C, with a dry season in spring and a heavy rainy season in summer. (3) Chavarrillo, El Lencero, Tuzamapan, and Vaqueria are between 800 m and 1000 m a.s.l.; the main vegetation is subevergreen forest, and the annual mean temperature is 20 °C. (4) San Isidro, Teocelo, Xalapa, and Xico are above 1100 m up to 1300 m; the original vegetation was topical montane cloud forest, however, deforestation has been rampant since the second half of the 20th century, and most of the original vegetation has been lost; the annual mean temperature is 18 °C, with light rains all year long and a heavy rainy season in summer. Field collections were conducted by direct sampling from nests using an aspirator. At each site, four different nests were identified, and a minimum of ten individuals per nest were collected. Nests were selected ad libitum; however, the selected nests were separated by at least 200 m to ensure that ant batches belonged to different nests. Specimens were preserved in 96% ethanol and stored at −20 °C until DNA extraction. The collected material was deposited at the Phylogenetic Systematics Laboratory (INECOL, Xalapa, Mexico). At least three individual from each nest were saved as voucher specimens.

### 2.2. Molecular Data

Fragments of two mitochondrial genes, cytochrome oxidase subunit I (*COI*) and cytochrome oxidase subunit II (*COII*), along with one nuclear long-wavelength rhodopsin (*LWRh*), were sequenced. Genomic DNA was extracted from a single individual (four to ten individuals were sequenced per nest) using a 5% (*w*/*v*) Chelex solution, following the protocol described by Singer-Sam et al. [17]. PCR amplification was conducted in a final volume of 20 μL, containing 5 μL of DNA template, 4 μL of 5X buffer, 2 μL of MgCl_2_ (25 mM), 2 μL of dNTPs (8 mM), 1 μL of each primer (10 mM), 0.2 μL of Taq polymerase (5 U/μL), and 5 μL of ddH_2_O. The PCR was conducted in Peltier effect thermocyclers applying specific conditions for each marker. For *COI*, the protocol consisted of 30 cycles of denaturing at 95 °C for 45 s, annealing at 45 °C for 30 s, and extension at 73 °C for 1 min; for *COII*, 30 cycles of denaturing at 95 °C for 45 s, annealing at 50 °C for 30 s, and extension at 73 °C for 1 min; and for *LWRh*, 35 cycles of denaturing at 95 °C for 45 s, annealing at 47 °C for 30 s, and extension at 73 °C for 1 min. For each protocol, a final extension cycle of 5 min at 73 °C was added. All PCR runs included positive and negative controls to detect potential contamination. Amplified PCR products were visualized in 1% agarose gels stained with GelRed^TM^ (Biotium, Fremont, CA, USA). Successful reactions were purified using the Gel and PCR Clean-up Kit (Macherey-Nagel, Düren, Germany) following the standard protocol indicated by the manufacturer. Finally, nucleotide sequencing was performed elsewhere by Macrogen Inc. (Seoul, South Korea).

### 2.3. Sequence Processing and Molecular Analyses

Raw sequences were assembled and edited in Sequencher v.5.2.4 (Gene Codes Corporation, Ann Arbor, MI, USA). Genetic variation and population structure were assessed using several statistical approaches. Haplotypes (H), haplotype diversity (Hd), nucleotide diversity (π), and segregating sites (S) were calculated following the method of Nei and Kumar [18] using DNASP v.6 [19]. Nucleotide composition and bias were calculated for each molecular marker, as well as the pair-wise Kimura 2-parameter corrected distances between collection sites. To estimate the fraction of total genetic variation distributed among nests, within and between collection sites, an analysis of molecular variance (AMOVA) was conducted using ARLEQUIN v.3.5 [20]. A total of 1000 permutations were performed to test the AMOVA statistical significance. Additionally, F_ST_ values for pairwise localities were calculated following the method of Lynch and Crease [21]. To identify genetically congruent geographic regions, a spatial analysis of molecular variance (SAMOVA) was performed using SAMOVA v.1.0 [22]. The number of groups (K) tested ranged from 2 to 13, and as suggested by Dupanloup et al. [22], the partition with the highest Φ_CT_ value was selected.

### 2.4. Phylogenetic and Network Analyses

Phylogenetic relationships were recovered under Bayesian inference using MrBayes v.3.2.3 [23]. Partitions from different molecular markers may contain incongruent phylogenetic signal due to differences in inheritance lines, effective population size, etc. Therefore, *COI*, *COII*, and *LWRh* markers were used simultaneously for phylogenetic analyses, but declared as unlinked partition. The starting best-fit nucleotide substitution model for each partition was determined using JMODELTEST v.2.02 [24] under the Akaike Information Criterion. Three different ant species (i.e., *Solenopsis invicta, Dorymyrmex insanus,* and *Dorymyrmex elegans*; sequences downloaded from GenBank, accretion numbers Si: AY950719, HQ215540; Di: AF147046; De: DQ353369, DQ353155) were included as outgroup for the purposes of rooting the tree. The Markov chains Monte Carlo were run for 50 million generations with four parallel chains, sampling every 1000 generations. The burn-in value was determined by the stabilization of log-likelihood values, and a majority-rule consensus tree was constructed to estimate posterior probabilities. Additionally, to visualize haplotype relationships, a minimum-spanning network was generated using POPART [25].

### 2.5. Divergence Time Estimation and Demographic History

We used the software BEAST v.1.10.5 [26] to estimate divergence times and historical demographic trends. The tree topology inferred from the Bayesian analysis was used as a prior. Calibrating a tree is difficult and involves making a number of assumptions. Unfortunately, there is a lack of fossil data for this ant species. Therefore, we decided to use a proposed mitochondrial rate of 1.7% s/s/my for the *COI* partition [27]. According to these authors, the *COII* evolves at a slower rate; therefore, for this partition we set the recommended substitution rate of 1.2% s/s/my. A relaxed uncorrelated lognormal clock model was implemented, with independent substitution rates estimated for each gene partition. A coalescent process was set as the prior for the tree model. The Markov chains Monte Carlo were run for 20 million generations, with sampling every 1000 generations. The analysis was repeated four times, and convergence was assessed using TRACER v.1.6 [28] to ensure that the effective sample sizes exceeded 200. The single runs were combined with LogCombiner implemented in the BEAST package. Trees were summarized using TreeAnnotator v.1.6.1, and displayed in FigTree v.1.3.1. To detect population expansion events, Fu’s F test [29] was estimated. To contrast the Fu’s F test results, a generalized Skyline-plot analysis was performed to assess for sudden bottleneck or expansion events. Also, a Mantel test was performed to evaluate potential isolation by distance among populations. Finally, gene flow between populations was estimated using the software Migrate v.5 [30]. This software estimates effective population sizes, past migration rates between n populations, assuming a migration matrix model with asymmetric migration rates and different subpopulation sizes. Each analysis was based on ten long chains and was run for a minimum of 10 million generations with a burn-in of 15% of the initial data.

## 3. Results

### 3.1. Genetic Diversity

The alignment of the mitochondrial *COI* resulted in a 679 bp fragment, corresponding to the second half of the gene, from position 766 to 1444 in the gene sequence of *Drosophila melanogaster* (GenBank accession number U37541). The 451 bp sequence fragment for the *COII* gene aligned with positions 30 to 492 in the *D. melanogaster* reference sequence (GenBank accession number KY559387). The amplified fragment for the nuclear *LWRh* was 383 bp in length. This fragment consisted of one intron sequence (87 bp) and regions of two flanking exons. Its identity was confirmed by multiple hits with NCBI nucleotide blast search in GenBank. The combined data set encompassed a full alignment of 1531 nucleotides. Pseudogenes originating from translocated fragments are among the main sources of error during sequence analyses. We are certain that the generated sequences are not pseudogenes based on the following evidence: (a) nonsense codons or frame shifts were not obtained; (b) all amino acid codons were translated using the insect genetic code without ambiguities or intermediate stop codons; and (c) the amino acid sequence of each marker is consistent with homologous fragments stored at GenBank. The nucleotide composition analysis of *COI* and *COII* revealed a composition bias in both genes (*COI*: 0.297, *COII*: 0.398; Supplementary Material Tables S1 and S2). Both mitochondrial markers have elevated thymine content (around 40%) and a reduced frequency of guanine (11% and 6% respectively). In contrast, the *LWRh* gene showed almost no nucleotide bias (0.033, Table S3). The uncorrected relative genetic distance among the *COI* haplotypes was, on average, 0.97%, while the Kimura 2-parameter corrected distance was, on average, 0.99% (Table S4). For *COII*, the uncorrected relative genetic distance among haplotypes was 0.76%, with a K2P-corrected distance of 0.77% (Table S5). The nuclear LWRh gene was highly conserved, and no nucleotide variation was detected.

The aligned sequences recovered 21 different haplotypes identified by a total of 42 segregating sites (*COI* 28, *COII* 14, *LWRh* 0; Table S6). The nests from a single location with the highest number of segregating sites were from Teocelo (S = 15), followed by Jalcomulco (S = 13; Table 2), whereas the lowest genetic diversity was found in the Vaqueria population, where all the sampled nests had the same haplotype (Hap_17; Table 3). In most populations, haplotype differences were due to transitions. The only exceptions were Apazapan and La Mancha (Table 2). Hap_01 and Hap_02, present at Apazapan, differed from each other at position 225 of the *COI*, where a mutation between adenine and thymine occurred. In La Mancha, the difference occurred between Hap_08 and Hap_12 at position 76 of the *COII*, with a single point mutation (G:C; Table S6). Globally, the average π was 0.0026, with the highest value of 0.0051 in Teocelo and the lowest in Vaqueria (π = 0; Table 2). An average of 2.5 haplotypes per locality were registered. The highest haplotype diversity was observed again in Teocelo, where each nest had its own haplotype (Table 3). All ants from a nest shared the same haplotype, except for one individual collected from La Mancha nest 01 and one from Xalapa nest 01. The individual from Xalapa exhibited a point mutation in the *COI* gene (position 451, A:G), whereas the one from La Mancha showed a point mutation in the COII gene (position 76, C:G). Fu’s F test suggests that historical demographic expansion is not significantly different from zero, indicating that the populations have remained demographically stable throughout time. Nonetheless, at a regional scale (i.e., all populations analyzed together) the Fu’s F test retrieved a value of −1.7, suggesting a possible demographic expansion. Finally, the AMOVA results indicate an incipient genetic structure with an F_ST_ value of 0.775 and show that the highest proportion of genetic variation (61%) occurs among groups (Table S7).

### 3.2. Interrelationships

**Figure 2:**
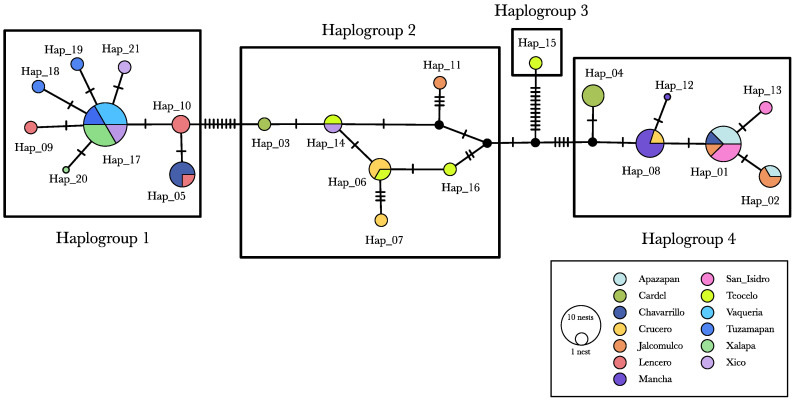
Haplotype network. Hap_15 was the most distant haplotype, differing by 14 point mutations from Hap_16, its closest haplotype. These haplotypes were found in Teocelo in nests 200 m apart from each other. Haplotypes 12 and 20 were recovered from a single ant each.

Four haplogroups were inferred from the haplotype network (Figure 2). Haplogroup 1, the most widespread, was primarily found in the localities of Chavarrillo, Tuzamapan, Vaquería, Xalapa, Xico, and Lencero. Haplogoup 1 was separated from Haplogroup 2 by eight point mutations. Haplogroup 2 comprised six recognized haplotypes and three missing haplotypes. Haplogroup 2 exhibited the greatest internal diversity, with an average of 2.3 mutational steps among its haplotypes. Haplogroup 3 was separated from Haplogroup 2 by eleven point mutations and consisted of a singleton (Hap_15) found in Teocelo nest 03. Finally, Haplogroup 4 was separated from Haplogroup 2 by four point mutations and included six haplotypes, primarily distributed among Apazapan, Jalcomulco, and Chavarrillo.

**Figure 3:**
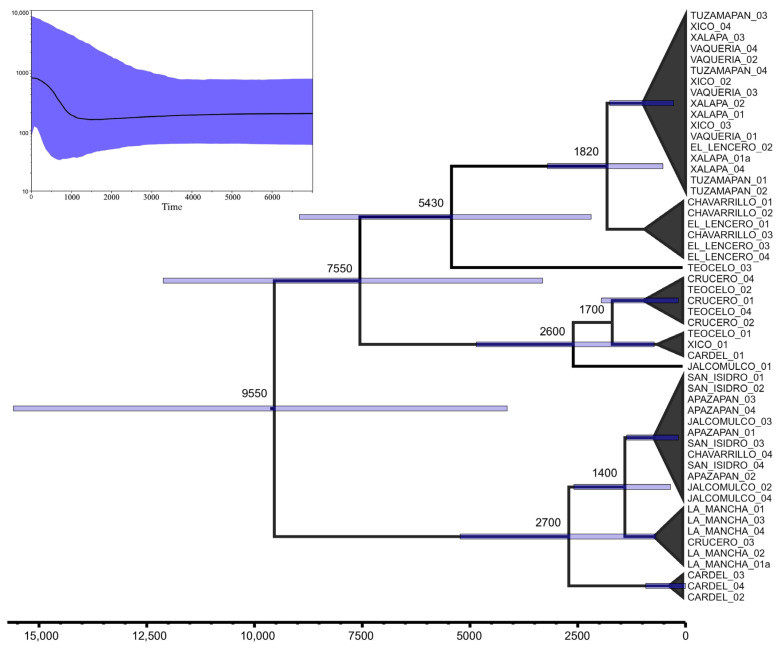
*Dorymyrmex bicolor* genealogy and chronogram. Outgroup branch has been pruned out from the tree for graphic purposes. Top left, skyline-plot showing a recent increment in the effective population size.

The genealogical structure of *Dorymyrmex bicolor* revealed two major clades (Figure 3). Broadly speaking, one of these clades could be associated with mountainous localities ranging from 700 to 1400 m above sea level (i.e., Xalapa, Xico, Teocelo, Tuzamapan, Vaquería, Chavarrillo, and El Lencero). However, three nests from the lowland locality El Crucero, situated at 132 m above sea level (Table 1), were recovered within this clade. The second clade, referred to as the “lowland clade”, encompassed localities located from 700 m to sea level. Nevertheless, all nests from San Isidro, a mountain locality located at 1100 m, were recovered within this lowland clade. Such incipient altitudinal structure was further disrupted by the presence of some nests from Chavarrillo, Jalcomulco, Cardel, and El Crucero, spread in both the mountain and lowland clades. According to our dating estimates, the radiation within the mountain clade began approximately 7500 years ago, while the radiation in the lowland clade started more recently, around 2700 years ago. Neither clade had nests from a single locality that were recovered as monophyletic. Even the four nests from Vaquería, which shared the same haplotype (Hap_17; Table 3), did not form an isolated unique geographic lineage, as Hap_17 is widely distributed in 12 different nests throughout four mountain localities (Vaqueria, Tuzamapan, Xalapa, and Xico). Local-scale differentiation (i.e., between geographic sites) appears to have occurred in recent times, within the last 1000 years (Figure 3).

### 3.3. Historical Demography

The SAMOVA (Table 4) identified four geographic–genetic groups. However, these groups did not fully correspond to the haplogroups identified in the haplotype network. The greatest coincide was observed between the SAMOVA Group I and the haplotype network Haplogroup 1 (Figure 2). Nonetheless, Teocelo was part of a different group (Group III), and some nests from Chavarrillo also fell outside of Group I. SAMOVA results indicate that nearly 70% of the total genetic variation was attributed to differences among the four geographic–genetic groups, whereas only 2.86% was explained by the variation among populations within groups. Although the genetic–geographic differentiation (F_CT_) was statistically significant, it accounted for 0.697 of the total variation (Table 4).

**Figure 4:**
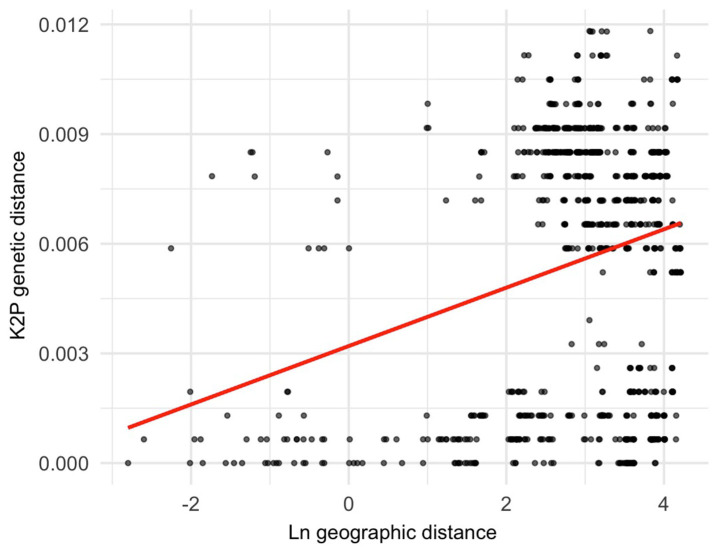
The Mantel test results indicate that the genetic distance has a direct relationship with the geographic distance. This pattern is consistent with a model of restricted gene flow.

The Mantel test (Figure 4) detected a weak but statistically significant correlation between genetic and geographic distances (R^2^ = 0.0097, *p* = 0.002), indicating limited gene flow across geographic space. Gene flow was detected among the four SAMOVA groups. The highest migration rate occurred from Group II to Group IV, with a total of 776 individuals per generation (Table 5). In contrast, the lowest migration was observed from Group III to Group I, with 78 individuals per generation, corresponding to the two most geographically distant groups [i.e., from Cardel (lowlands, 22 m) to Chavarrillo, El Lencero, Tuzamapan, Vaqueria, Xalapa, and Xico (mountains above 800 m)]. Reciprocal migration among groups is notably asymmetric; for example, while 776 individuals per generation migrated from Group II to Group IV, only 183 individuals moved in the opposite direction. The most “symmetric” migratory exchange was calculated between Groups I and III (135 individuals from I to III, and 78 individuals from III to I). Overall, there appears to be a more intense gene flow from mountainous areas to lowlands than in the opposite direction (Table 5).

Pairwise F_ST_ values between collection sites (Table S8) revealed a wide range of genetic differentiation. The lowest differentiation was found among populations from Tuzamapan, Xalapa, and Xico. Conversely, the highest average differentiation was observed between La Mancha and San Isidro and the rest of the localities. The absence of genetic differentiation is inversely related to the high gene flow among the localities of Vaquería, Xalapa, and Xico, all of which belong to genetic–geographic Group I identified by SAMOVA. Alternatively, the high differentiation may be correlated with the geographic distance between La Mancha and San Isidro and the rest of the localities. Finally, the skyline-plot reconstruction (Figure 3) suggests that the effective population size has been stable over time, with a sudden increase only occurring during the last 1000 years.

## 4. Discussion

The genetic diversity analysis for *Dorymyrmex bicolor* populations revealed an incipient hierarchical structure. Mitochondrial markers identified a total of 21 haplotypes. A lot of variation was observed within populations, with between 1 and 4 different haplotypes found. The haplotype diversity could be considered as moderate, with an average of 0.6 (standard deviation from 0 to 0.27) per population, whereas the average nucleotide diversity could be considered as low (π = 0.0026; standard deviation from 0 to 0.0036). Although the overall Fu’s F test is consistent with demographic stability and the Mantel test showed only a weak correlation between genetic differentiation and geographic distance (R^2^ = 0.0097), the AMOVA detected genetic differentiation among regional groups (F_ST_ = 0.775), with 61.5% of the variance attributed to among-group differences. Four haplogroups were inferred, yet nests within a locality were not monophyletic, and haplotypes were often shared across sites. These patterns may reflect a combination of reproductive and dispersal dynamics, although specific studies addressing such mechanisms in *D. bicolor* remain scarce.

In *Dorymyrmex*, alate queens and males are known to participate in nuptial flights [16], a common dispersal strategy in ants that facilitates gene flow and the colonization of new habitats. While both monogyny and polygyny have been reported within the genus *Dorymyrmex* [31], our results support a predominantly monogynous colony structure in *D. bicolor*. This is based on the lack of genetic variation in the nuclear *LWRh* gene and the presence of a single mitochondrial haplotype within each nest. Such a pattern is consistent with a parental system, where a single queen typically founds and maintains the colony. The distinction between monogynous and polygynous systems is relevant when interpreting colony founding strategies: while polygynous species often expand via budding, a process associated with limited dispersal and strong local structure [32], monogynous species usually rely on independent aerial dispersal, which can promote higher gene flow and lower genetic differentiation [33]. However, the relationship between monogyny and genetic structure is not always straightforward [34]. Although traditional expectations associate monogyny with panmixia and minimal local differentiation, studies such as that by Sundström et al. [35] on *Formica exsecta* have documented limited female dispersal and significant genetic subdivision even in monogynous species.

One possible explanation for the incipient genetic–geographic structure observed in *D. bicolor* populations in Central Veracruz is the species’ presumed high dispersal capacity via nuptial flights [16,33]. While no detailed data on queen number are available for the bicolor pyramid ant, the presence of alate reproductives and ecological traits shared with other dispersive ants suggest that nuptial flights may contribute to gene flow across geographic areas. In *Brachymyrmex patagonicus*, a predominantly monogynous ant species, the absence of isolation by distance has been linked to high dispersal capacity and extensive gene flow [32]. Although *D. bicolor* does not demonstrate a total absence of isolation by distance, the observed low but significant correlation between genetic and geographic distances (R^2^ = 0.0097, *p* = 0.002) supports the idea of effective, yet not unlimited, dispersal. Such dispersal likely helps maintain genetic similarity between populations, disrupting the spatial genetic structure that would be expected under more limited movement.

In addition to dispersal via nuptial flights, the ecological traits of *D. bicolor* may further facilitate gene flow. The species is known to thrive in a wide range of environments [16], and its presence in both lowland and montane habitats across Veracruz suggests a degree of ecological flexibility. Such adaptability may promote colonization across fragmented or ecologically similar sites, enhancing connectivity at the landscape scale. This could contribute to the weak genetic structuring observed at the local level. Nevertheless, the Mantel test suggests that gene flow is still somewhat restricted by geographic distance. This may be due to the interruption of connectivity across ecological or topographic barriers, or to past divergence events that left traces of genetic structure in this lineage.

In addition to ecological and behavioral traits, historical abiotic processes might explain the recovered genetic patterns. On a broader phylogeographic scale, high dispersal ability is a common trait across the genus *Dorymyrmex*. Phylogenomic studies have revealed a long history of active dispersal across the New World, including multiple recolonization events in the Caribbean [16]. Although these findings are relevant to continental scales, they suggest that similar processes may operate regionally. As our data suggest, if *D. bicolor* shows similar patterns, ongoing gene flow might prevent the formation of clear geographic structure, even in the complex landscape of Central Veracruz. Historical geological processes may have contributed to structure the genetic divergence observed in this ant species. The split between the mountain and lowland clades in Central Veracruz aligns temporally with significant volcanic and tectonic events during the Late Holocene [36,37]. Divergence time estimates, approximately 7500 years ago for the mountain clade and 2700 years ago for the lowland clade, coincide broadly with known eruptive episodes in the region. The older radiation date of the mountain clade overlaps with the Holocene eruption of the Citlaltépetl volcano (~8500–9000 years ago), which produced extensive pyroclastic flows (Citlaltépetl ignimbrite) and significantly altered the landscape of the Sierra Madre Oriental [36]. These events may have contributed to the emergence of altitudinal or ecological barriers that reduced gene flow, thereby promoting phylogeographic divergence.

The more recent radiation of the lowland clade (~2700 years ago) is temporally closer to the Texmola eruptive episode (~2000 years ago), which was marked by block-and-ash flows south of Citlaltépetl [38]. These events may have influenced regional habitat configuration. Additionally, volcanic activity in the Xalapa monogenetic volcanic field, which includes Late Pleistocene to Holocene pyroclastic deposits and lava flows, likely shaped local topography through the formation of ravines and fault systems [39]. Although there is no direct evidence linking the Xalapa volcanic field to the genetic patterns in *D. bicolor*, its geological features may have acted as geographic barriers, limiting ecological connectivity. These processes offer a likely abiotic explanation for the genetic structure observed in *D. bicolor*.

This historical scenario is consistent with patterns reported in other invertebrates. For example, *Falagonia mexicana* (Coleoptera: Staphylinidae) exhibits strong phylogeographic structure associated with recent volcanic activity and topographic complexity in the Trans-Mexican Volcanic Belt [9], while long-term isolation in “sky islands” has led to deep cryptic divergence in species such as the harvestman *Sclerobunus robustus*, despite minimal morphological differentiation [40]. A similar process may underlie the divergence observed among *D. bicolor* haplogroups, which appear to reflect geographic and altitudinal partitioning across a volcanically dynamic landscape. However, genetic structure in *D. bicolor* does not follow a uniform pattern across spatial scales. While genetic differentiation is observed between the mountain and lowland clades, differentiation at finer geographic scales is subtler. Localities such as Xalapa, Xico, and Vaquería exhibit low pairwise F_ST_ values and evidence of high gene flow, suggesting effective dispersal across short distances. These differences may result from variation in historical connectivity or habitat continuity.

Altitudinal separation may also have been further reinforced by Holocene climatic fluctuations that reduced habitat connectivity in Central Veracruz. During the Middle and Late Holocene, shifts in the position of the Intertropical Convergence Zone and regional climatic instability led to episodes of prolonged drought across Mesoamerica [41]. Notably, the droughts of the Late Classic period (~600–900) and the Little Ice Age (~1350–1850) are well documented to have impacted vegetation structure, hydrology, and overall ecosystem productivity (*e.g*., decreased riparian vegetation and lowered lake levels) [9,42]. These environmental changes likely disrupted habitat continuity between zones, which may have reduced ant population sizes and limited movement between mountain and lowland areas. Although there is no direct evidence of population collapse in *D. bicolor*, the timing of these climatic events matches the recent divergence of highland and lowland clades. This could explain the shared haplotypes between nearby but ecologically different sites. Altogether, these patterns support the idea that past climate events temporarily blocked gene flow and strengthened isolation between populations at different elevations.

*Dorymyrmex* species are often regarded as generalists in both habitat use and foraging behavior [16]. *Dorymyrmex bicolor*, like other species of the genus, has been frequently reported in open and disturbed habitats, such as roadsides and degraded soils [43], suggesting ecological flexibility. This may facilitate dispersal across heterogeneous landscapes, as observed in generalist carabid beetles that maintain genetic connectivity across fragmented environments, in contrast to more specialized species [44]. Furthermore, while fine-scale structure in insects is sometimes linked to inbreeding [45]. Unfortunately, data on inbreeding are not yet available for this species; nonetheless, the observed genetic patterns do not suggest strong isolation at the local scale.

Finally, we must acknowledge that the taxonomic complexity of *D. bicolor* may complicate the interpretation of spatial genetic patterns. The species has been described as polyphyletic, meaning that populations currently assigned to this taxon might represent a species complex [16]. This could result in masking the phylogeographic structure. In this context, Teocelo nest 3 (Hap_15) stands out due to its large genetic differentiation. This haplotype differs by 11 point mutations from the closest haplogroups. Its uniqueness and deep divergence suggest it could be a separate cryptic lineage, supporting the idea that *D. bicolor* may include multiple evolutionary lineages. The incipient phylogenetic structure recovered, however, is also consistent with a pattern of incomplete lineage sorting. Many haplotypes found in a specific geographic place are more closely related to haplotypes distributed in a different locality. For instance, Hap_03 identified in nest 01 from Cardel is closely related to Hap_14 discovered in nest 01 from Xico. On the one hand, these haplotypes are differentiated from each other by a single point mutation, despite their nests being separated by a linear distance of 70 km. On the other hand, Hap_03 differs by 11 point mutations from Hap_04 recovered in another nest sampled at Cardel, even though these nests were located less than 200 m from each other.

Our results suggest that the genetic structure of *D. bicolor* in Central Veracruz reflects a combination of historical climatic and geological processes, broad ecological tolerance, and dispersal. These patterns raise key questions about the species’ evolutionary history across its full geographic range. Future research should aim to include populations from other parts of its distribution, northern Mexico, the southwestern United States and South America, to determine whether the patterns observed here are consistent across regions or simply reflect local dynamics. Comparative phylogeographic studies that integrate nuclear markers, ecological data, and environmental modeling will be essential to assess the role of topographic barriers, climatic gradients, and past volcanic activities in shaping genetic diversity throughout the species’ range. These studies could also help determine whether the bicolor pyramid ant is a single species or a group of distinct, possibly cryptic, regional lineages.

## Figures and Tables

**Table 1:** Collection sites.

Site	Nest	Latitude	Longitude	Altitude	Habitat
Apazapan	01	19.3187	−96.7159	476 m	Evergreen forest and oak woodland
	02	19.3195	−96.7169		
	03	19.3206	−96.7192		
	04	19.3254	−96.7332		
Cardel	01	19.3712	−96.3763	22 m	Subdeciduous tropical forest.
	02	19.3682	−96.3825		
	03	19.3646	−96.3828		
	04	19.3661	−96.3799		
Chavarrillo	01	19.4228	−96.7857	811 m	Foothill subevergreen forest
	02	19.4229	−96.7927		
	03	19.4226	−96.7872		
	04	19.4202	−96.7860		
El Crucero	01	19.3170	−96.5223	132 m	Low deciduous forest
	02	19.3167	−96.5267		
	03	19.3155	−96.5278		
	04	19.3155	−96.5268		
Jalcomulco	01	19.3296	−96.7656	418 m	Subdeciduous tropical forest in river
	02	19.3292	−96.7628		canyon
	03	19.3286	−96.7574		
	04	19.3325	−96.7650		
El Lencero	01	19.4912	−96.8182	1042 m	Cloud forest and secondary oak
	02	19.4871	−96.8183		woodlands
	03	19.4875	−96.8163		
	04	19.4897	−96.8155		
La Mancha	01	19.5903	−96.3800	0 m	Mangroves, coastal dunes
	02	19.5903	−96.3793		
	03	19.5915	−96.3793		
	04	19.5962	−96.3773		
San Isidro	01	19.3625	−96.9046	1116 m	Montane cloud forest
	02	19.6053	−96.5421		
	03	19.6036	−96.9014		
	04	19.6064	−96.8998		
Teocelo	01	19.3817	−96.9812	1187 m	Montane cloud forest
	02	19.3961	−96.9824		
	03	19.3902	−96.9572		
	04	19.3902	−96.9572		
Tuzamapan	01	19.3882	−96.8760	869 m	Montane cloud and subevergreen
	02	19.3945	−96.8689		forest
	03	19.3979	−96.8711		
	04	19.3969	−96.8642		
Vaquería	01	19.4138	−96.8367	864 m	Montane cloud and subevergreen
	02	19.4143	−96.8369		forest
	03	19.4142	−96.8389		
	04	19.4049	−96.8349		
Xalapa	01	19.5121	−96.9431	1355 m	Montane cloud forest
	02	19.5125	−96.9452		
	03	19.5127	−96.9440		
	04	19.5128	−96.9456		
Xico	01	19.4307	−97.0142	1351 m	Montane cloud forest
	02	19.4286	−97.0032		
	03	19.4027	−96.9935		
	04	19.4086	−96.9897		

Altitude is measured in meters above sea level.

**Table 2:** Descriptors of genetic diversity.

Population	n	S	Ts	Tv	π	SD π	H	Hd	SD Hd	Fu’s F	*p*
Apazapan	4	1	0	1	0.0003	0.0004	2	0.50	0.27	0.172	0.33
Cardel	4	9	9	0	0.0029	0.0022	2	0.50	0.27	3.777	0.18
Chavarrillo	4	13	12	1	0.0042	0.0030	2	0.50	0.27	4.604	0.12
El Crucero	4	12	12	0	0.0039	0.0028	3	0.83	0.22	1.792	0.43
Jalcomulco	4	12	10	2	0.0040	0.0029	3	0.83	0.22	1.835	0.42
Lencero	4	3	3	0	0.0010	0.0009	3	0.83	0.22	−0.288	0.34
La Mancha	4	1	0	1	0.0003	0.0003	2	0.40	0.24	0.090	0.29
San Isidro	4	1	1	0	0.0003	0.0004	2	0.50	0.27	0.172	0.34
Teocelo	4	15	15	0	0.0051	0.0036	4	1.00	0.18	0.043	0.31
Tuzamapan	4	2	2	0	0.0006	0.0006	3	0.83	0.22	−0.887	0.25
Vaquería	4	0	0	0	0.0000	0.0000	1	0.00	0.00	NA	NA
Xalapa	4	1	1	0	0.0003	0.0003	2	0.40	0.24	0.090	0.39
Xico	4	11	11	0	0.0036	0.0026	3	0.83	0.22	1.655	0.44
Global	52	42	39	3	0.0055	0.0003	21	0.92	0.022	−1.732	0.06

n: Nests per site, S: segregated sites, Ts: transitions, Tv: transversions, π: nucleotide diversity, SD: standard deviation, H: haplotypes, Hd: haplotype diversity.

**Table 3:** Haplotype frequency and distribution.

	Apa	Car	Cha	Cru	Jal	Len	Man	SIs	Teo	Tuz	Vaq	Xal	Xic	Nests
Hap_01	3	–	1	–	1	–	–	3	–	–	–	–	–	8
Hap_02	1	–	–	–	2	–	–	–	–	–	–	–	–	3
Hap_03	–	1	–	–	–	–	–	–	–	–	–	–	–	1
Hap_04	–	3	–	–	–	–	–	–	–	–	–	–	–	3
Hap_05	–	–	3	–	–	1	–	–	–	–	–	–	–	4
Hap_06	–	–	–	2	–	–	–	–	1	–	–	–	–	3
Hap_07	–	–	–	1	–	–	–	–	–	–	–	–	–	1
Hap_08	–	–	–	1	–	–	4	–	–	–	–	–	–	5
Hap_09	–	–	–	–	–	1	–	–	–	–	–	–	–	1
Hap_10	–	–	–	–	–	2	–	–	–	–	–	–	–	2
Hap_11	–	–	–	–	1	–	–	–	–	–	–	–	–	1
Hap_12	–	–	–	–	–	–	1	–	–	–	–	–	–	1
Hap_13	–	–	–	–	–	–	–	1	–	–	–	–	–	1
Hap_14	–	–	–	–	–	–	–	–	1	–	–	–	1	2
Hap_15	–	–	–	–	–	–	–	–	1	–	–	–	–	1
Hap_16	–	–	–	–	–	–	–	–	1	–	–	–	–	1
Hap_17	–	–	–	–	–	–	–	–	–	2	4	4	2	12
Hap_18	–	–	–	–	–	–	–	–	–	1	–	–	–	1
Hap_19	–	–	–	–	–	–	–	–	–	1	–	–	–	1
Hap_20	–	–	–	–	–	–	–	–	–	–	–	1	–	1
Hap_21	–	–	–	–	–	–	–	–	–	–	–	–	1	1

**Table 4:** Spatial analysis of molecular variance.

Variation Source	DF	Sum of Squares	Variance Components	% of Variation
Among groups	3	143.5	3.85 *	69.72
Populations within groups	9	19.6	0.16 *	2.86
Within populations	41	62.1	1.51 *	27.42
Total	53	225.1	5.52 *	

F_SC_ = 0.094 *, F_ST_ = 0.726 *, F_CT_ = 0.697 *; * Significance < 0.0001. Recovered groups: I (Chavarrillo, El Lencero, Tuzamapan, Vaqueria, Xalapa, Xico), II (Apazapan, Jalcomulco, La Mancha, San Isidro), III (El Crucero, Teocelo), IV (Cardel).

**Table 5:** Migrant exchange between the four groups recovered by the SAMOVA.

To/From	Group I	Group II	Group III	Group IV
Group I	−	326	78	183
Group II	231	−	238	183
Group III	135	459	−	268
Group IV	545	776	666	−

## Data Availability

All DNA sequences generated during this study are deposited and available in GenBank under the following accession numbers: *COI* PV844383 (Hap_01), PV844384 (Hap_02), PV844385 (Hap_03), PV844386 (Hap_04, 05, 06), PV844387 (Hap_05, 10), PV844388 (Hap_06, 16), PV844389 (Hap_07), PV8443890 (Hap_09, 17, 21), PV844391 (Hap_11), PV8443892 (Hap_13), PV844393 (Hap_14), PV844394 (Hap_15), PV844395 (Hap_18), PV844396 (Hap_19), and PV844397 (Hap_20); *COII* PV855120 (Hap_01, 02, 08, 13), PV855121 (Hap_03, 06, 07, 14), PV855122 (Hap_04), PV855123 (Hap_05), PV855124 (Hap_09), PV855125 (Hap_10, 17, 18, 19, 20), PV855126 (Hap_11), PV855127 (Hap_12), PV855128 (Hap_15), PV855129 (Hap_16), PV855130 (Hap_21), and *LWRh* PV855131 (all haplotypes).

## Supplementary Materials

The following supporting information can be downloaded at https://www.mdpi.com/article/10.3390/insects16080785/s1: Figure S1. *Dorymyrmex bicolor* nest entrance; Table S1: *COI* nucleotide composition and bias; Table S2: *COII* nucleotide composition and bias; Table S3: *LWRh* nucleotide composition and bias; Table S4: *COI* pairwise distances; Table S5: *COII* pairwise distances; Table S6: Haplotype segregated sites; Table S7: Analysis of molecular variance; Table S8: Pairwise genetic differentiation and gene flow between collection sites.

## Abbreviations

The following abbreviations are used in this manuscript:

AMOVA	Analysis of Molecular Variance
*COI*	*Cytochrome Oxidase Subunit I*
*COII*	*Cytochrome Oxidase Subunit II*
*LWRh*	*Long-wavelength Rhodopsin*
SAMOVA	Spatial Analysis of Molecular Variance
