# Supplementary material for: The Great Wanderer: The Phylogeographic History of the Bicolor Pyramid Ant (Dorymyrmex bicolor Wheeler, 1906) in Central Veracruz, Mexico
Source: Insects. 2025 Jul 31;16(8):785. doi: 10.3390/insects16080785 (PMC12386616; doi:10.3390/insects16080785)
Supplement: Supplementary file 1 [file insects-16-00785-s001.zip › insects-3763579-supplementary.pdf]

SUPPLEMENTARY MATERIAL

The Great Wanderer: The Phylogeographic History of the Bicolor Pyramid Ant (*Dorymyrmex bicolor* Wheeler, 1906) in Central Veracruz, Mexico

María Gómez-Lazaga and Alejandro Espinosa de los Monteros

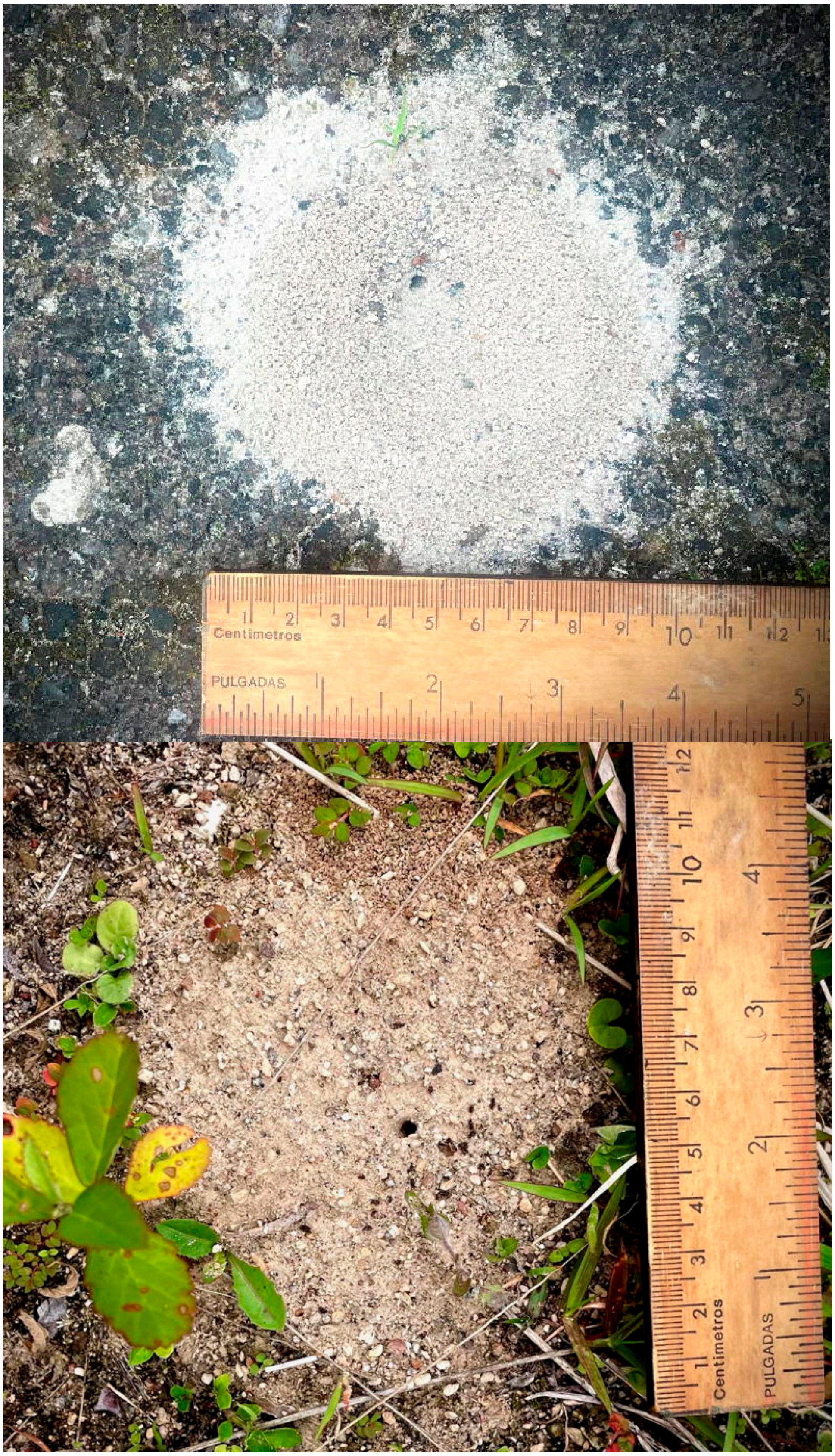

Figure S1. *Dorymyrmex bicolor* nest entrance.

Table S1. *COI* nucleotide composition and bias.

Nest	A	C	G	T
Apazapan 01	0.29844	0.18388	0.11740	0.40028
Apazapan 02	0.29703	0.18388	0.11740	0.40170
Apazapan 03	0.29844	0.18388	0.11740	0.40028
Apazapan 04	0.29844	0.18388	0.11740	0.40028
Cardel 01	0.29986	0.18529	0.11740	0.39745
Cardel 02	0.29986	0.18388	0.11740	0.39887
Cardel 03	0.29986	0.18388	0.11740	0.39887
Cardel 04	0.29986	0.18388	0.11740	0.39887
Chavarrillo 01	0.30127	0.18529	0.11598	0.39745
Chavarrillo 02	0.30127	0.18529	0.11598	0.39745
Chavarrillo 03	0.30127	0.18529	0.11598	0.39745
Chavarrillo 04	0.29844	0.18388	0.11740	0.40028
El Crucero 01	0.29986	0.18529	0.11740	0.39745
El Crucero 02	0.29844	0.18529	0.11881	0.39745
El Crucero 03	0.29986	0.18388	0.11740	0.39887
El Crucero 04	0.29986	0.18529	0.11740	0.39745
El Lencero 01	0.30127	0.18529	0.11598	0.39745
El Lencero 02	0.30127	0.18388	0.11598	0.39887
El Lencero 03	0.30127	0.18529	0.11598	0.39745
El Lencero 04	0.30127	0.18529	0.11598	0.39745
Jalcomulco 01	0.29844	0.18529	0.11881	0.39745
Jalcomulco 02	0.29703	0.18388	0.11740	0.40170
Jalcomulco 03	0.29844	0.18388	0.11740	0.40028
Jalcomulco 04	0.29703	0.18388	0.11740	0.40170
La Mancha 01	0.29986	0.18388	0.11740	0.39887
La Mancha 01a	0.29986	0.18388	0.11740	0.39887
La Mancha 02	0.29986	0.18388	0.11740	0.39887
La Mancha 03	0.29986	0.18388	0.11740	0.39887
La Mancha 04	0.29986	0.18388	0.11740	0.39887
San Isidro 01	0.29844	0.18388	0.11740	0.40028
San Isidro 02	0.29844	0.18388	0.11740	0.40028
San Isidro 03	0.29844	0.18388	0.11740	0.40028
San Isidro 04	0.29844	0.18246	0.11740	0.40170
Teocelo 01	0.29844	0.18529	0.11881	0.39745
Teocelo 02	0.29986	0.18529	0.11740	0.39745
Teocelo 03	0.29844	0.18529	0.11881	0.39745
Teocelo 04	0.29986	0.18529	0.11740	0.39745
Tuzamapan 01	0.30127	0.18388	0.11598	0.39887
Tuzamapan 02	0.29986	0.18388	0.11740	0.39887
Tuzamapan 03	0.30127	0.18388	0.11598	0.39887
Tuzamapan 04	0.30127	0.18246	0.11598	0.40028
Vaqueria 01	0.30127	0.18388	0.11598	0.39887
Vaqueria 02	0.30127	0.18388	0.11598	0.39887
Vaqueria 03	0.30127	0.18388	0.11598	0.39887
Vaqueria 04	0.30127	0.18388	0.11598	0.39887
Xalapa 01	0.30127	0.18388	0.11598	0.39887
Xalapa 01a	0.29986	0.18388	0.11740	0.39887
Xalapa 02	0.30127	0.18388	0.11598	0.39887
Xalapa 03	0.30127	0.18388	0.11598	0.39887
Xalapa 04	0.30127	0.18388	0.11598	0.39887
Xico 01	0.29844	0.18529	0.11881	0.39745
Xico 02	0.30127	0.18388	0.11598	0.39887
Xico 03	0.30127	0.18388	0.11598	0.39887
Xico 04	0.30127	0.18388	0.11598	0.39887
Mean	0.29988	0.18424	0.11698	0.39889

Nucleotide bias = 0.297

Table S2. *COII* nucleotide composition and bias.

Nest	A	C	G	T
Apazapan 01	0.33481	0.17073	0.06430	0.43016
Apazapan 02	0.33481	0.17073	0.06430	0.43016
Apazapan 03	0.33481	0.17073	0.06430	0.43016
Apazapan 04	0.33481	0.17073	0.06430	0.43016
Cardel 01	0.33481	0.17073	0.06430	0.43016
Cardel 02	0.33481	0.16630	0.06430	0.43459
Cardel 03	0.33481	0.16630	0.06430	0.43459
Cardel 04	0.33481	0.16630	0.06430	0.43459
Chavarrillo 01	0.33481	0.17517	0.06430	0.42572
Chavarrillo 02	0.33481	0.17517	0.06430	0.42572
Chavarrillo 03	0.33481	0.17517	0.06430	0.42572
Chavarrillo 04	0.33481	0.17073	0.06430	0.43016
El Crucero 01	0.33481	0.17073	0.06430	0.43016
El Crucero 02	0.33481	0.17073	0.06430	0.43016
El Crucero 03	0.33481	0.17073	0.06430	0.43016
El Crucero 04	0.33481	0.17073	0.06430	0.43016
El Lencero 01	0.33481	0.17517	0.06430	0.42572
El Lencero 02	0.33481	0.17517	0.06430	0.42572
El Lencero 03	0.33481	0.17295	0.06430	0.42794
El Lencero 04	0.33481	0.17295	0.06430	0.42794
Jalcomulco 01	0.33259	0.16851	0.06652	0.43237
Jalcomulco 02	0.33481	0.17073	0.06430	0.43016
Jalcomulco 03	0.33481	0.17073	0.06430	0.43016
Jalcomulco 04	0.33481	0.17073	0.06430	0.43016
La Mancha 01	0.33481	0.17073	0.06430	0.43016
La Mancha 01a	0.33481	0.16851	0.06652	0.43016
La Mancha 02	0.33481	0.17073	0.06430	0.43016
La Mancha 03	0.33481	0.17073	0.06430	0.43016
La Mancha 04	0.33481	0.17073	0.06430	0.43016
San Isidro 01	0.33481	0.17073	0.06430	0.43016
San Isidro 02	0.33481	0.17073	0.06430	0.43016
San Isidro 03	0.33481	0.17073	0.06430	0.43016
San Isidro 04	0.33481	0.17073	0.06430	0.43016
Teocelo 01	0.33481	0.17073	0.06430	0.43016
Teocelo 02	0.33481	0.17073	0.06430	0.43016
Teocelo 03	0.33259	0.17295	0.06652	0.42794
Teocelo 04	0.33481	0.16851	0.06430	0.43237
Tuzamapan 01	0.33481	0.17295	0.06430	0.42794
Tuzamapan 02	0.33481	0.17295	0.06430	0.42794
Tuzamapan 03	0.33481	0.17295	0.06430	0.42794
Tuzamapan 04	0.33481	0.17295	0.06430	0.42794
Vaqueria 01	0.33481	0.17295	0.06430	0.42794
Vaqueria 02	0.33481	0.17295	0.06430	0.42794
Vaqueria 03	0.33481	0.17295	0.06430	0.42794
Vaqueria 04	0.33481	0.17295	0.06430	0.42794
Xalapa 01	0.33481	0.17295	0.06430	0.42794
Xalapa 01a	0.33481	0.17295	0.06430	0.42794
Xalapa 02	0.33481	0.17295	0.06430	0.42794
Xalapa 03	0.33481	0.17295	0.06430	0.42794
Xalapa 04	0.33481	0.17295	0.06430	0.42794
Xico 01	0.33481	0.17073	0.06430	0.43016
Xico 02	0.33481	0.17517	0.06430	0.42572
Xico 03	0.33481	0.17295	0.06430	0.42794
Xico 04	0.33481	0.17295	0.06430	0.42794
Mean	0.33473	0.17159	0.06442	0.42925

Nucleotide bias = 0.398

Table S3. *LWRh* nucleotide composition and bias.

Nest	A	C	G	T
Apazapan 01	0.24543	0.24282	0.24021	0.27154
Apazapan 02	0.24543	0.24282	0.24021	0.27154
Apazapan 03	0.24543	0.24282	0.24021	0.27154
Apazapan 04	0.24543	0.24282	0.24021	0.27154
Cardel 01	0.24543	0.24282	0.24021	0.27154
Cardel 02	0.24543	0.24282	0.24021	0.27154
Cardel 03	0.24543	0.24282	0.24021	0.27154
Cardel 04	0.24543	0.24282	0.24021	0.27154
Chavarrillo 01	0.24543	0.24282	0.24021	0.27154
Chavarrillo 02	0.24543	0.24282	0.24021	0.27154
Chavarrillo 03	0.24543	0.24282	0.24021	0.27154
Chavarrillo 04	0.24543	0.24282	0.24021	0.27154
El Crucero 01	0.24543	0.24282	0.24021	0.27154
El Crucero 02	0.24543	0.24282	0.24021	0.27154
El Crucero 03	0.24543	0.24282	0.24021	0.27154
El Crucero 04	0.24543	0.24282	0.24021	0.27154
El Lencero 01	0.24543	0.24282	0.24021	0.27154
El Lencero 02	0.24543	0.24282	0.24021	0.27154
El Lencero 03	0.24543	0.24282	0.24021	0.27154
El Lencero 04	0.24543	0.24282	0.24021	0.27154
Jalcomulco 01	0.24543	0.24282	0.24021	0.27154
Jalcomulco 02	0.24543	0.24282	0.24021	0.27154
Jalcomulco 03	0.24543	0.24282	0.24021	0.27154
Jalcomulco 04	0.24543	0.24282	0.24021	0.27154
La Mancha 01	0.24543	0.24282	0.24021	0.27154
La Mancha 01a	0.24543	0.24282	0.24021	0.27154
La Mancha 02	0.24543	0.24282	0.24021	0.27154
La Mancha 03	0.24543	0.24282	0.24021	0.27154
La Mancha 04	0.24543	0.24282	0.24021	0.27154
San Isidro 01	0.24543	0.24282	0.24021	0.27154
San Isidro 02	0.24543	0.24282	0.24021	0.27154
San Isidro 03	0.24543	0.24282	0.24021	0.27154
San Isidro 04	0.24543	0.24282	0.24021	0.27154
Teocelo 01	0.24543	0.24282	0.24021	0.27154
Teocelo 02	0.24543	0.24282	0.24021	0.27154
Teocelo 03	0.24543	0.24282	0.24021	0.27154
Teocelo 04	0.24543	0.24282	0.24021	0.27154
Tuzamapan 01	0.24543	0.24282	0.24021	0.27154
Tuzamapan 02	0.24543	0.24282	0.24021	0.27154
Tuzamapan 03	0.24543	0.24282	0.24021	0.27154
Tuzamapan 04	0.24543	0.24282	0.24021	0.27154
Vaqueria 01	0.24543	0.24282	0.24021	0.27154
Vaqueria 02	0.24543	0.24282	0.24021	0.27154
Vaqueria 03	0.24543	0.24282	0.24021	0.27154
Vaqueria 04	0.24543	0.24282	0.24021	0.27154
Xalapa 01	0.24543	0.24282	0.24021	0.27154
Xalapa 01a	0.24543	0.24282	0.24021	0.27154
Xalapa 02	0.24543	0.24282	0.24021	0.27154
Xalapa 03	0.24543	0.24282	0.24021	0.27154
Xalapa 04	0.24543	0.24282	0.24021	0.27154
Xico 01	0.24543	0.24282	0.24021	0.27154
Xico 02	0.24543	0.24282	0.24021	0.27154
Xico 03	0.24543	0.24282	0.24021	0.27154
Xico 04	0.24543	0.24282	0.24021	0.27154
Mean	0.24543	0.24282	0.24021	0.27154

Nucleotide bias = 0.033

Table S4. *COI* pairwise distances. K2P above, and relative uncorrected below diagonal.

	Apa 01	Apa 02	Car 01	Car 02	Cha 01	Cru 01	Cru 02	Len 02	Jal 01	Ma 01a	S.I 04	Teo 01	Teo 03	Tuz 02	Tuz 04	Vaq 01	Xal 01a	Xal 04	Xic 01
Apazapan 01	—	0.0014	0.0085	0.0014	0.0129	0.0085	0.0129	0.0143	0.0071	0.0014	0.0014	0.0071	0.0187	0.0158	0.0158	0.0143	0.0158	0.0143	0.0071
Apazapan 02	0.0014	—	0.0100	0.0028	0.0143	0.0100	0.0143	0.0158	0.0085	0.0028	0.0028	0.0085	0.0202	0.0172	0.0172	0.0158	0.0172	0.0158	0.0085
Cardel 01	0.0085	0.0099	—	0.0071	0.0071	0.0028	0.0071	0.0086	0.0043	0.0071	0.0100	0.0014	0.0129	0.0100	0.0100	0.0086	0.0100	0.0086	0.0014
Cardel 02	0.0014	0.0028	0.0071	—	0.0114	0.0071	0.0114	0.0129	0.0057	0.0000	0.0028	0.0057	0.0173	0.0143	0.0143	0.0129	0.0143	0.0129	0.0057
Chavarrillo 01	0.0127	0.0141	0.0071	0.0113	—	0.0100	0.0143	0.0014	0.0114	0.0114	0.0143	0.0086	0.0173	0.0028	0.0028	0.0014	0.0028	0.0014	0.0086
El Crucero 01	0.0085	0.0099	0.0028	0.0071	0.0099	—	0.0043	0.0114	0.0043	0.0071	0.0100	0.0014	0.0129	0.0129	0.0129	0.0114	0.0129	0.0114	0.0014
El Crucero 02	0.0127	0.0141	0.0071	0.0113	0.0141	0.0042	—	0.0158	0.0086	0.0114	0.0143	0.0057	0.0173	0.0173	0.0173	0.0158	0.0173	0.0158	0.0057
El Lencero 02	0.0141	0.0156	0.0085	0.0127	0.0014	0.0113	0.0156	—	0.0129	0.0129	0.0158	0.0100	0.0187	0.0014	0.0014	0.0000	0.0014	0.0000	0.0100
Jalcomulco 01	0.0071	0.0085	0.0042	0.0057	0.0113	0.0042	0.0085	0.0127	—	0.0057	0.0085	0.0028	0.0143	0.0143	0.0143	0.0129	0.0143	0.0129	0.0028
La Mancha 01a	0.0014	0.0028	0.0071	0.0000	0.0113	0.0071	0.0113	0.0127	0.0057	—	0.0028	0.0057	0.0173	0.0143	0.0143	0.0129	0.0143	0.0129	0.0057
San Isidro 04	0.0014	0.0028	0.0099	0.0028	0.0141	0.0099	0.0141	0.0156	0.0085	0.0028	—	0.0085	0.0202	0.0172	0.0172	0.0158	0.0172	0.0158	0.0085
Teocelo 01	0.0071	0.0085	0.0014	0.0057	0.0085	0.0014	0.0057	0.0099	0.0028	0.0057	0.0085	—	0.0114	0.0114	0.0114	0.0100	0.0114	0.0100	0.0000
Teocelo 03	0.0184	0.0198	0.0127	0.0170	0.0170	0.0127	0.0170	0.0184	0.0141	0.0170	0.0198	0.0113	—	0.0202	0.0202	0.0187	0.0202	0.0187	0.0114
Tuzamapan 02	0.0156	0.0170	0.0099	0.0141	0.0028	0.0127	0.0170	0.0014	0.0141	0.0141	0.0170	0.0113	0.0198	—	0.0028	0.0014	0.0028	0.0014	0.0114
Tuzamapan 04	0.0156	0.0170	0.0099	0.0141	0.0028	0.0127	0.0170	0.0014	0.0141	0.0141	0.0170	0.0113	0.0198	0.0028	—	0.0014	0.0028	0.0014	0.0114
Vaqueria 01	0.0141	0.0156	0.0085	0.0127	0.0014	0.0113	0.0156	0.0000	0.0127	0.0127	0.0156	0.0099	0.0184	0.0014	0.0014	—	0.0014	0.0000	0.0100
Xalapa 01a	0.0156	0.0170	0.0099	0.0141	0.0028	0.0127	0.0170	0.0014	0.0141	0.0141	0.0170	0.0113	0.0198	0.0028	0.0028	0.0014	—	0.0014	0.0114
Xalapa 04	0.0141	0.0156	0.0085	0.0127	0.0014	0.0113	0.0156	0.0000	0.0127	0.0127	0.0156	0.0099	0.0184	0.0014	0.0014	0.0000	0.0014	—	0.0100
Xico 01	0.0071	0.0085	0.0014	0.0057	0.0085	0.0014	0.0057	0.0099	0.0028	0.0057	0.0085	0.0000	0.0113	0.0113	0.0113	0.0099	0.0113	0.0099	—

Table S5. *COII* pairwise distances. K2P above, and relative uncorrected below diagonal.

	Apa 01	Car 01	Car 02	Cha 01	Cru 04	Len 02	Jal 01	Man 01a	S.I. 01	Teo 03	Teo 04	Tuz 01	Vaq 01	Xal 01a	Xic 02
Apazapan 01	—	0.0089	0.0045	0.0089	0.0089	0.0089	0.0135	0.0022	0.0000	0.0089	0.0067	0.0067	0.0067	0.0067	0.0089
Cardel 01	0.0089	—	0.0089	0.0089	0.0000	0.0089	0.0045	0.0112	0.0089	0.0135	0.0022	0.0067	0.0067	0.0067	0.0089
Cardel 02	0.0044	0.0089	—	0.0089	0.0089	0.0089	0.0135	0.0067	0.0045	0.0089	0.0067	0.0067	0.0067	0.0067	0.0089
Chavarrillo 01	0.0089	0.0089	0.0089	—	0.0089	0.0045	0.0135	0.0112	0.0089	0.0089	0.0112	0.0022	0.0022	0.0022	0.0045
El Crucero 04	0.0089	0.0000	0.0089	0.0089	—	0.0089	0.0045	0.0112	0.0089	0.0135	0.0022	0.0067	0.0067	0.0067	0.0089
El Lencero 02	0.0089	0.0089	0.0089	0.0044	0.0089	—	0.0135	0.0112	0.0089	0.0089	0.0112	0.0022	0.0022	0.0022	0.0045
Jalcomulco 01	0.0133	0.0044	0.0133	0.0133	0.0044	0.0133	—	0.0157	0.0135	0.0181	0.0067	0.0112	0.0112	0.0112	0.0135
La Mancha 01a	0.0022	0.0111	0.0067	0.0111	0.0111	0.0111	0.0155	—	0.0022	0.0112	0.0089	0.0089	0.0089	0.0089	0.0112
San Isidro 01	0.0000	0.0089	0.0044	0.0089	0.0089	0.0089	0.0133	0.0022	—	0.0089	0.0067	0.0067	0.0067	0.0067	0.0089
Teocelo 03	0.0089	0.0133	0.0089	0.0089	0.0133	0.0089	0.0177	0.0111	0.0089	—	0.0112	0.0067	0.0067	0.0067	0.0089
Teocelo 04	0.0067	0.0022	0.0067	0.0111	0.0022	0.0111	0.0067	0.0089	0.0067	0.0111	—	0.0089	0.0089	0.0089	0.0112
Tuzamapan 01	0.0067	0.0067	0.0067	0.0022	0.0067	0.0022	0.0111	0.0089	0.0067	0.0067	0.0089	—	0.0000	0.0000	0.0022
Vaqueria 01	0.0067	0.0067	0.0067	0.0022	0.0067	0.0022	0.0111	0.0089	0.0067	0.0067	0.0089	0.0000	—	0.0000	0.0022
Xalapa 01a	0.0067	0.0067	0.0067	0.0022	0.0067	0.0022	0.0111	0.0089	0.0067	0.0067	0.0089	0.0000	0.0000	—	0.0022
Xico 02	0.0089	0.0089	0.0089	0.0044	0.0089	0.0044	0.0133	0.0111	0.0089	0.0089	0.0111	0.0022	0.0022	0.0022	—

Table S6. Haplotype segregated sites.

	Cytochrome C subunit I																												*Cytochrome C subunit II*													
			1	1	2	2	2	2	2	2	3	3	4	4	4	4	4	4	4	5	5	5	5	5	6	6	6	6				1	1	1	1	1	1	2	3	3	3	3
		1	1	8	1	2	2	3	5	8	5	8	0	5	7	8	8	9	9	0	0	1	1	9	0	7	7	9	4	7	7	2	2	3	4	5	7	8	1	2	4	9
	9	5	4	6	3	2	5	1	9	2	7	7	2	1	1	6	9	5	8	4	7	0	6	4	3	2	5	2	5	5	6	0	6	2	4	6	4	8	8	7	5	0
Hap_01	a	g	t	c	c	a	a	a	c	c	t	t	c	a	a	a	t	t	t	a	g	g	c	a	c	g	t	g	t	t	c	t	t	t	c	a	c	a	c	t	t	c
Hap_02	.	.	.	.	.	.	t	.	.	.	.	.	.	.	.	.	.	.	.	.	.	.	.	.	.	.	.	.	.	.	.	.	.	.	.	.	.	.	.	.	.	.
Hap_03	.	.	.	.	.	.	.	g	.	.	.	.	.	.	g	.	.	a	c	.	a	a	.	.	.	.	.	.	.	c	.	.	.	.	.	.	t	.	t	c	.	.
Hap_04	.	.	.	.	.	.	.	.	.	.	.	.	.	.	.	.	.	a	.	.	.	.	.	.	.	.	.	.	.	.	.	.	.	.	.	.	t	.	.	.	.	t
Hap_05	.	.	.	.	t	.	.	g	.	.	.	c	.	.	.	.	c	a	c	.	a	a	.	.	t	.	.	.	.	c	.	.	c	.	.	.	t	.	.	.	c	.
Hap_06	.	.	.	.	.	.	.	g	.	.	.	.	.	.	g	.	.	a	c	.	a	.	.	.	.	a	.	.	.	c	.	.	.	.	.	.	t	.	t	c	.	.
Hap_07	.	.	c	.	.	.	.	g	.	t	.	.	.	.	g	g	.	a	c	.	a	.	.	.	.	a	.	.	.	c	.	.	.	.	.	.	t	.	t	c	.	.
Hap_08	.	.	.	.	.	.	.	.	.	.	.	.	.	.	.	.	.	a	.	.	.	.	.	.	.	.	.	.	.	.	.	.	.	.	.	.	.	.	.	.	.	.
Hap_09	.	.	.	.	t	.	.	g	.	.	.	c	.	.	.	.	c	a	c	.	a	a	t	.	t	.	.	.	c	c	.	.	c	.	.	.	t	.	.	.	.	.
Hap_10	.	.	.	.	t	.	.	g	.	.	.	c	.	.	.	.	c	a	c	.	a	a	.	.	t	.	.	.	.	c	.	.	c	.	.	.	t	.	.	.	.	.
Hap_11	.	.	.	.	.	.	.	.	.	.	.	.	.	.	g	.	.	a	c	g	a	.	.	.	.	.	.	.	.	c	.	.	.	.	t	g	t	.	t	c	.	.
Hap_12	.	.	.	.	.	.	.	.	.	.	.	.	.	.	.	.	.	a	.	.	.	.	.	.	.	.	.	.	.	.	g	.	.	.	.	.	.	.	.	.	.	.
Hap_13	.	.	.	.	.	.	.	.	.	.	.	.	t	.	.	.	.	.	.	.	.	.	.	.	.	.	.	.	.	.	.	.	.	.	.	.	.	.	.	.	.	.
Hap_14	.	.	.	.	.	.	.	g	.	.	.	.	.	.	g	.	.	a	c	.	a	.	.	.	.	.	.	.	.	c	.	.	.	.	.	.	t	.	t	c	.	.
Hap_15	g	a	.	.	t	g	.	g	t	.	c	.	.	.	g	.	.	a	c	.	a	.	.	.	.	.	c	a	.	.	.	.	c	c	.	.	t	g	.	.	.	.
Hap_16	.	.	.	.	.	.	.	g	.	.	.	.	.	.	g	.	.	a	c	.	a	.	.	.	.	a	.	.	.	.	.	.	.	.	.	.	t	.	t	c	.	.
Hap_17	.	.	.	.	t	.	.	g	.	.	.	c	.	.	.	.	c	a	c	.	a	a	t	.	t	.	.	.	.	c	.	.	c	.	.	.	t	.	.	.	.	.
Hap_18	.	.	.	.	t	.	.	g	.	.	.	c	.	.	.	.	c	a	c	.	a	a	t	g	t	.	.	.	.	c	.	.	c	.	.	.	t	.	.	.	.	.
Hap_19	.	.	.	t	t	.	.	g	.	.	.	c	.	.	.	.	c	a	c	.	a	a	t	.	t	.	.	.	.	c	.	.	c	.	.	.	t	.	.	.	.	.
Hap_20	.	.	.	.	t	.	.	g	.	.	.	c	.	g	.	.	c	a	c	.	a	a	t	.	t	.	.	.	.	c	.	.	c	.	.	.	t	.	.	.	.	.
Hap_21	.	.	.	.	t	.	.	g	.	.	.	c	.	.	.	.	c	a	c	.	a	a	t	.	t	.	.	.	.	c	.	c	c	.	.	.	t	.	.	.	.	.

Table S7. Analysis of molecular variance.

Variation Source	DF	Sum of squares	Variance components	% of variation
Among groups	1	106.8	3.79*	61.5
Populations within groups	11	55.9	0.86*	14.0
Within populations	41	61.9	1.51*	24.5
Total	53	224.8	8.16*	

F_{st} =0.775*; * = Significance < 0.001.

Table S8. Pairwise genetic differentiation and gene flow between collection sites. Fixation index below diagonal (F_{st}), migrants per generation above diagonal (M).

Location	Apazapan	Cardel	Chavarrillo	El Crucero	El Lencero	Jalcomulco	La Mancha	San Isidro	Teocelo	Tuzamapan	Vaqueria	Xalapa	Xico
Apazapan	—	0.5000	0.2692	0.2954	0.0417	Inf	0.2208	Inf	0.2841	0.0289	0.0096	0.0170	0.1579
Cardel	**0.5000**	—	0.6667	1.4000	0.1875	2.9091	0.6670	0.5000	1.0882	0.1528	0.1250	0.1183	0.4762
Chavarrillo	0.6500	0.4286	—	0.6410	16.0000	0.6600	0.2441	0.2692	0.7414	1.2500	1.0833	0.9002	12.0000
El Crucero	**0.6286**	0.2632	0.4382	—	0.2344	1.1967	0.3004	0.2954	Inf	0.1944	0.1667	0.1528	0.6571
El Lencero	**0.9231**	**0.7273**	0.0303	**0.6809**	—	0.1983	0.0395	0.0417	0.3182	1.2500	0.7500	0.8199	14.0000
Jalcomulco	-0.0256	0.1467	0.4307	0.2947	**0.7160**	—	1.7268	10.0000	0.8750	0.1680	0.1445	0.1326	0.4294
La Mancha	**0.6937**	**0.4285**	**0.6720**	**0.6247**	**0.9268**	**0.2245**	—	0.2208	0.2739	0.0273	0.0095	0.0167	0.1495
San Isidro	0.0000	**0.5000**	0.6500	**0.6286**	**0.9231**	0.0476	**0.6937**	—	0.2841	0.0288	0.0096	0.0170	0.1579
Teocelo	**0.6377**	0.3148	0.4028	-0.0641	**0.6111**	0.3636	**0.6461**	**0.6377**	—	0.2650	0.2350	0.2098	0.8696
Tuzamapan	**0.9455**	**0.7660**	**0.2857**	**0.7200**	0.2857	**0.7485**	**0.9482**	**0.9455**	**0.6536**	—	Inf	19.1667	Inf
Vaqueria	**0.9811**	**0.8000**	**0.3158**	**0.7500**	0.4000	**0.7758**	**0.9813**	**0.9811**	**0.6803**	0.0000	—	Inf	Inf
Xalapa	**0.9671**	**0.8087**	**0.3571**	**0.7659**	**0.3788**	**0.7904**	**0.9677**	**0.9671**	**0.7044**	0.0254	-0.0526	—	8.8726
Xico	**0.7600**	0.5122	0.0400	0.4321	0.0345	**0.5380**	**0.7698**	**0.7600**	0.3651	0.0000	0.0000	0.0534	—

Fst significant values in bold, significance level = 0.05
